# Integrative Proteome and Phosphoproteome Profiling of Early Cold Response in Maize Seedlings

**DOI:** 10.3390/ijms23126493

**Published:** 2022-06-10

**Authors:** Jiayun Xing, Jinjuan Tan, Hanqian Feng, Zhongjing Zhou, Min Deng, Hongbing Luo, Zhiping Deng

**Affiliations:** 1 College of Agronomy, Hunan Agricultural University, Changsha 410128, China; jyxing11@126.com (J.X.); hdengmin@163.com (M.D.); 2 State Key Laboratory for Managing Biotic and Chemical Threats to the Quality and Safety of Agro-Products, Institute of Virology and Biotechnology, Zhejiang Academy of Agricultural Sciences, Hangzhou 310021, China; donger1103@163.com (J.T.); hanqianfeng@foxmail.com (H.F.); zj_20020101@163.com (Z.Z.)

**Keywords:** maize, cold stress, TMT-labeling, proteome, phosphoproteome, seedlings, photosynthesis, spliceosome

## Abstract

Cold limits the growth and yield of maize in temperate regions, but the molecular mechanism of cold adaptation remains largely unexplored in maize. To identify early molecular events during cold shock, maize seedlings were treated under 4 °C for 30 min and 2 h, and analyzed at both the proteome and phosphoproteome levels. Over 8500 proteins and 19,300 phosphopeptides were quantified. About 660 and 620 proteins were cold responsive at protein abundance or site-specific phosphorylation levels, but only 65 proteins were shared between them. Functional enrichment analysis of cold-responsive proteins and phosphoproteins revealed that early cold response in maize is associated with photosynthesis light reaction, spliceosome, endocytosis, and defense response, consistent with similar studies in *Arabidopsis*. Thirty-two photosynthesis proteins were down-regulated at protein levels, and 48 spliceosome proteins were altered at site-specific phosphorylation levels. Thirty-one kinases and 33 transcriptional factors were cold responsive at protein, phosphopeptide, or site-specific phosphorylation levels. Our results showed that maize seedlings respond to cold shock rapidly, at both the proteome and phosphoproteome levels. This study provides a comprehensive landscape at the cold-responsive proteome and phosphoproteome in maize seedlings that can be a significant resource to understand how C_4_ plants respond to a sudden temperature drop.

## 1. Introduction

Cold temperature is a major environmental factor that limits the adaptation and growth of maize in temperate regions [1]. As a typical C_4_ plant originated in tropical regions, maize (*Zea mays* L.) has higher optimal growth temperature than C_3_ plants, and is more sensitive to cold stress especially during seed germination and early seedling growth stages [1,2,3,4]. Increasing the cold tolerance is a major breeding goal for maize breeders, as it is a bottleneck to reach the high potential of maize production in temperate areas [5].

Cold tolerance in plants is a complex quantitative polygenic trait, and has been extensively studied at the physiological, molecular, biochemical, and genetic levels, especially in the model plants *Arabidopsis* and rice [6,7,8,9,10]. Progress has been made in deciphering the signal transduction pathway of cold stress in *Arabidopsis* and rice [8,9]. It is generally accepted that cold stress leads to decreased cell membrane fluidity and a reorganized cytoskeleton, followed by a transient flux of calcium ions into the cytoplasm before triggering a cascade of molecular events [8,9]. At the molecular level, C-repeat/DREB binding factors (CBFs) act as key cold-response transcription factors that induce the expression of cold-regulated (*COR*) genes, after an induction by another transcription factor, Inducer of CBF expression1 (ICE1) [8,11,12,13]. Although similar signal perception and transduction pathways of cold response are shared between *Arabidopsis* and rice, there are likely distinct players or pathways in each species [8]. For example, rice Chilling-tOLerance Divergence 1 (COLD1) protein interacts with G-protein α subunit 1 (RGA1) and perceives the cold signal to trigger the calcium influx and activation of expression of *COR* genes [14], but rice COLD1 is distinct from its *Arabidopsis* orthologs AtGTG1 and AtGTG2 in intrinsic GTPase activity [14,15], and no implication of AtGTG1 and AtGTG2 in cold signaling has been reported so far [16].

Much progress has been made in understanding the physiological response of cold stress in maize [1,3,17], but the genetic basis of the cold adaptation in maize remains poorly characterized [1,3,17,18]. Physiological and genetic studies suggest that chilling temperatures affect distinct metabolic functions at different maize growth stages, each under the control of independent gene sets [1,17]. Temperatures below 10 °C negatively affect gemination rate and slow seedling establishment [1,19]. During the seedling growth stage, chilling temperatures reduce chlorophyll content, decrease photosynthesis rate including photosystem II efficiency, retard growth, and induce oxygen species that would cause cellular and tissue damages [1,3,17]. Genetic mapping and GWAS analyses have identified a large number of quantitative trait loci (QTL) and single nucleotide polymorphisms (SNPs) associated with distinct traits of cold tolerance [3,17], and have uncovered a few underlying candidate genes with mostly minor effects on chilling tolerance [1,4,5]. Large scale RNA-seq analyses have identified thousands of genes responsive to cold stress in maize [3,20,21,22,23], but functional characterization of cold-responsive genes was limited, and genetic verification of implication of candidate genes in cold tolerance was mostly performed in *Arabidopsis* and tobacco [3]. However, recently, Zeng et al. [18] reported that a mitogen-activated protein kinase ZmMPK8 phosphorylates a type-A Response Regulator 1 (ZmRR1) after cold stress, and showed that ZmMPK8 and ZmRR1 act as a negative and a positive regulator of cold tolerance in maize, respectively, using over-expression and CRISPR/Cas9-edited maize lines of each gene. The same group also showed that basic leucine zipper (bZIP) transcription factor bZIP68 acts as a negative regulator of chilling tolerance in maize by repressing expression of *COR* genes using mutant maize lines [24].

Reversible protein phosphorylation is a key regulatory mechanism in transducing the cold signal in plants [8,25]. For example, a plasma membrane protein CaM-regulated receptor-like kinase (CRLK) is activated by cold-activated Ca^2+^ influx and activates a MAPK signal cascade [26,27], leading to the modulation of ICE stability and *CBF* expression [28,29]. Rapid progress in the throughput and sensitivity of proteome and phosphoproteome profiling techniques has provided proteomics with an alternative tool in identifying cold tolerance regulators in plants, such as a maltose-metabolizing enzyme Disproportionating Enzyme 2 (DPE2) [30], and MAPK cascade proteins [28]. Cold-induced subcellular translocation of DPE2 is involved in the rapid accumulation of maltose that acts as compatible solute to protect cells from cold damage [30], while the activation of different members of MAPKs is critical for the induction or attenuation of the expression of *CBF* genes [28]. Recently, two large-scale phosphoproteome profiling studies on early cold response in *Arabidopsis* (within 2 h of cold shock) have provided significant resources to understand early molecular events during a sudden temperature drop in plants [31,32]. In this study, we have performed a multiplex isobaric tandem mass tags (TMT)-based quantitative proteomics approach to uncover the early cold signaling events in maize, and we have identified similarities and differences in early cold response in maize, when compared to previous similar studies in *Arabidopsis*.

## 2. Results

### 2.1. Proteome and Phosphoproteome Analyses of Maize Seedlings upon Short Time Cold Stress

**Figure 1:**
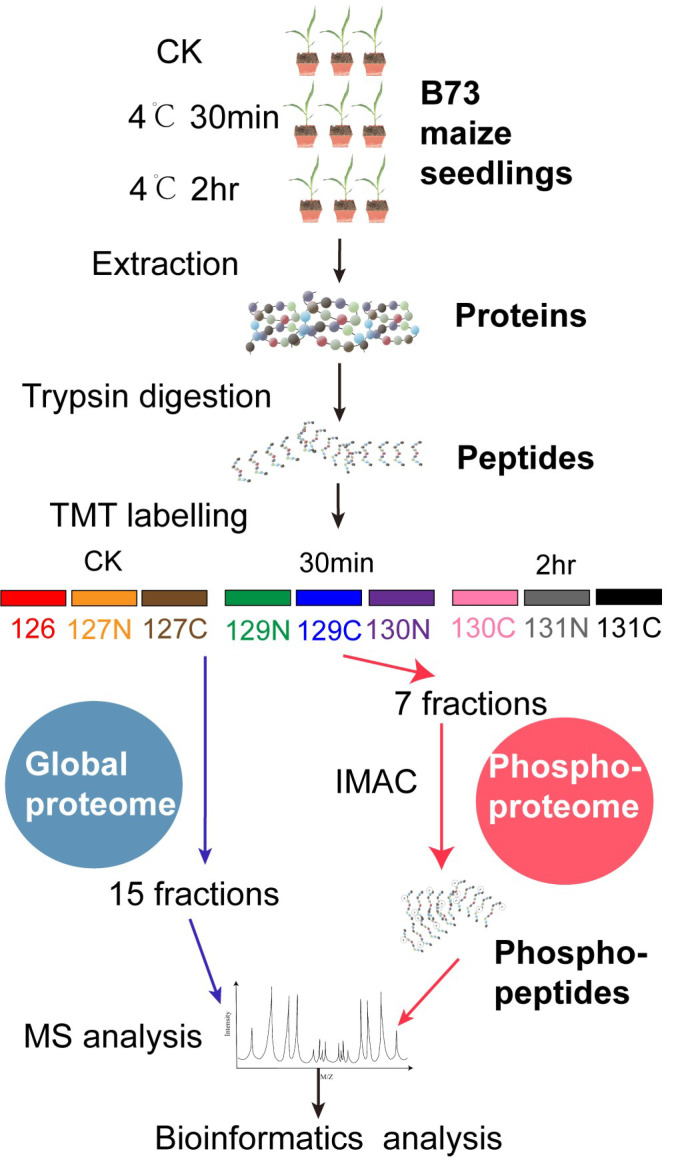
Workflow for global proteome and phosphoproteome profiling of maize seedlings under rapid temperature drop. Five-leaf-stage B73 maize seedlings were cold treated at 4 °C for 30 min and 2 h. Protein samples were labeled with individual TMT reagents, combined, and fractionated into 15 fractions for global proteome analysis or 7 fractions for phosphoproteome analysis.

To identify early cold-responsive proteins and phosphopeptides, maize seedlings were cold-treated at 4 °C for 30 min or 2 h, and multiplexed isobaric tandem mass tags (TMT)-based quantitative profiling was performed on the global proteome and phosphoproteome of the maize seedlings (Figure 1). In the global (or total) proteome, 8885 proteins were identified at the FDR cutoff of 1%, and 8567 proteins were quantified across the nine samples (each three biological repeats from the three time points, and proteins with missing values were discarded) (Table S1). In this workflow, phosphopeptides were enriched using Ga^3+^-based immobilized metal ion affinity chromatography [32]. In the phosphoproteome analysis, a total of 21,641 phosphopeptides (defined as phosphopeptide isoforms in Proteome Discoverer 2.4) ascribed to 5632 proteins were identified (Table S1), and the majority of phosphopeptides (18,342 or 84.8%) featured a single p-site (Table S1). A total of 19,320 phosphopeptides corresponding to 4803 proteins were quantified across the nine samples (Table S1), and 16,604 phosphopeptides corresponding to 14,125 phosphosites had a high confidence phosphosite with ptmRS site probability over 75% (Table S1), of which phosphorylation was situated primarily on serine (89.2%), and to a lesser extent, on threonine (10.5%) or tyrosine residue (0.24%).

**Figure 2:**
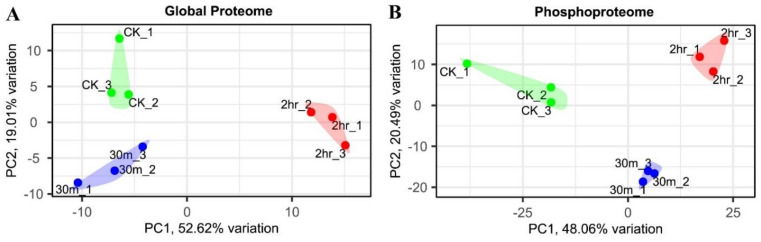
PCA analysis of the global proteome (**A**) and phosphoproteome (**B**) samples. The control samples (CK), the 30 min, and the 2 h samples are well separated on the PCA plots in both the global proteome and the phosphoproteome analyses.

Principal components analysis (PCA) was carried out to evaluate the variability of multiplexed global proteome and phosphoproteome samples. The three time-point groups (control, 30 min, and 2 h) were separated well in the PCA plots in both the global proteome and phosphoproteome analyses, suggesting reproducible differences present among the different groups (Figure 2). Interestingly, in the global proteome analysis, the control samples and the 30 min samples were mostly separated by the second component of the PCA plot, while in the phosphoproteome analysis, the control samples and the 30 min samples were separated by both the first and the second components of the PCA plots. These results suggest that the phosphoproteome responds more quickly than the global proteome, in agreement with similar studies in *Arabidopsis* [31,32].

### 2.2. Cold-Responsive Proteins and Phosphopeptides

**Figure 3:**
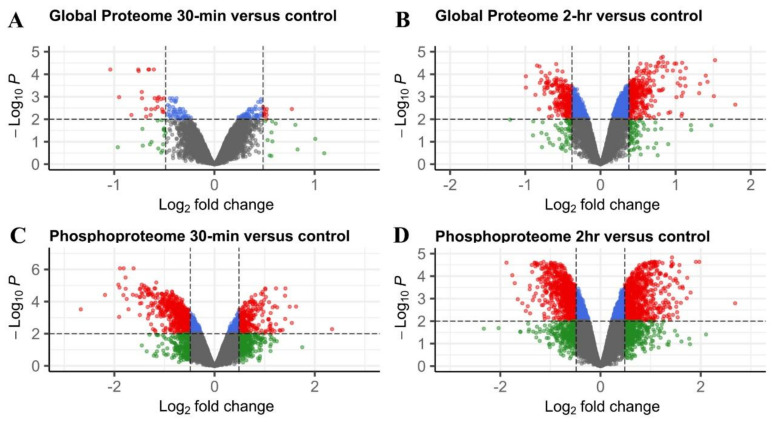
Volcano plot visualization of cold-responsive proteins (**A**,**B**) and phosphopeptides (**C**,**D**) under 30 min (**A**,**C**) or 2 h (**B**,**D**) of cold treatment. Cold-responsive proteins or phosphopeptides are depicted in red. X-axis is the Log2 of fold change (treatment /control) and Y-axis is the negative Log10 of the *p* value for independent t-test adjusted by the Benjamini–Hochberg procedure. Vertical dashed lines denote a fold change cutoff in either direction. The horizontal dashed line represents a cutoff of an adjusted *p* value of 0.01.

**Figure 4:**
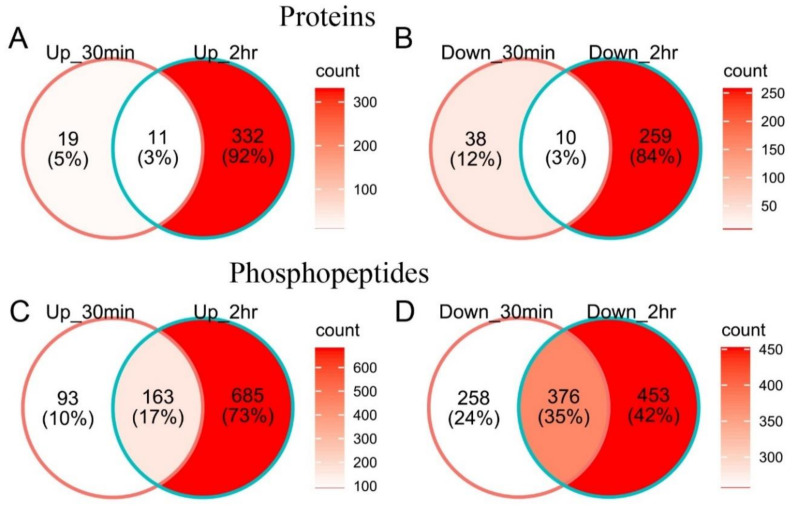
Venn diagram showing numbers of cold-responsive proteins (**A**,**B**) and phosphopeptides (**C**,**D**) of maize seedlings under different cold-shock durations (30 min and 2 h). Numbers of up-regulated (**A**,**C**) and down-regulated (**B**,**D**) ones were listed separately. Color in each region is coded according to the count of observations contained therein.

Technical and biological variations were considered when setting the threshold of significantly changed proteins [33,34]; a protein was considered cold responsive after 30 min or 2 h of cold treatment when the protein fold change > 1.3 or <0.77 and had an adjusted *p*-value of less than 0.01 when compared to the control, considering 99% of the intra-group coefficient of variation in global proteome was less than 30%. Based on the two criteria, a total of 78 cold-responsive proteins were detected after 30 min of cold treatment, including 30 up-regulated and 48 down-regulated ones (Figure 3 and Figure 4, Table S2). There were 612 cold-regulated proteins after 2 h of cold treatment in maize seedlings, 343 (56.0%) of which were up-regulated and 269 (44.0%) were down-regulated (Figure 3 and Figure 4, Table S2).

A phosphopeptide with a fold-change of >1.4 or <0.71 and an adjusted *p*-value of less than 0.01 was considered cold responsive, since the variance within each time-point group was slightly larger in the phosphopeptide samples (99% of the intra-group coefficient of variation was less than 40%). A total of 890 cold-responsive phosphopeptides were detected upon 30 min of cold stimuli, of which 256 (28.8%) phosphopeptides were up-regulated and 634 (71.2%) were down-regulated (Figure 3 and Figure 4, Table S2). There were 1677 cold-responsive phosphopeptides observed in maize seedlings after 2 h of cold treatment, 848 (50.6%) of which increased and 829 (49.4%) decreased in abundance (Figure 3 and Figure 4, Table S2). 

Interestingly, there were more down-regulated proteins than up-regulated proteins observed after 30 min of cold treatment, but more up-regulated proteins after 2 h of cold treatment. The same pattern held true for phosphopeptides; there were more down-regulated phosphopeptides at 30 min while slightly more up-regulated ones in 2 h. These observations were consistent with those observed in phosphopeptides in the early cold response of *Arabidopsis* seedlings [32]; however, only 7 out of 6733 *Arabidopsis* proteins (about 1%) were observed with altered protein accumulation, even after 2 h of cold treatment, indicating that the response in the global proteome in maize is much more rapid and pronounced than that in *Arabidopsis*.

**Figure 5:**
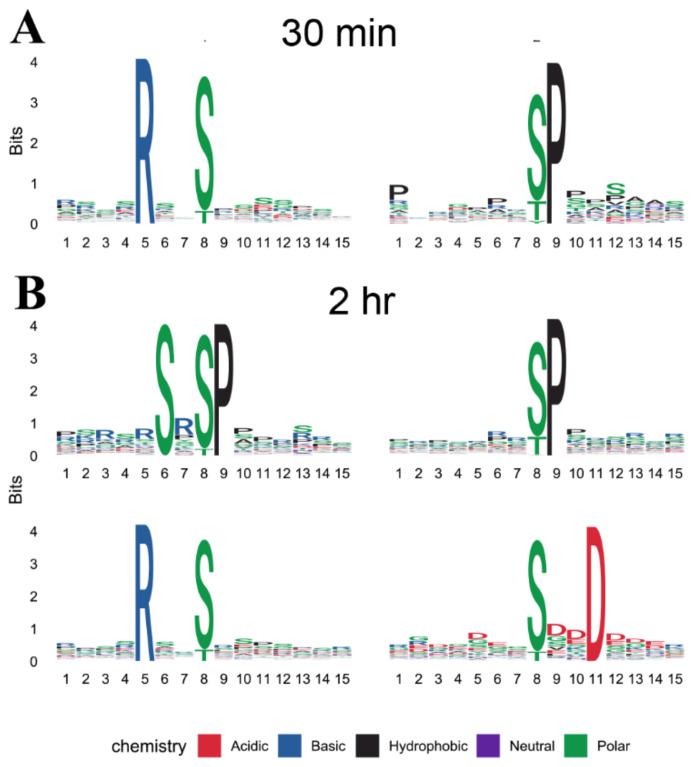
Motif analysis of cold up-regulated phosphopeptides. (**A**) Significantly enriched phosphorylation motifs of maize B73 under 30 min of cold stress. (**B**) Significantly enriched phosphorylation motifs of maize B73 under 2 h of cold stress.

Motif analysis was performed on cold-responsive phosphosites to predict the associated kinases. Two and four motifs were significantly enriched from cold up-regulated phosphopeptides after 30 min and 2 h of cold treatment, respectively (Figure 5), using the MoMo algorithm [35]. RXXS and SP motifs were enriched in both time points, while SXSP and SXXD motifs were additionally observed after 2 h of cold treatment. The RXXS or [RXXpS/pT] motif is an extremely common motif targeted by SNF1-related kinase II (SnRK2), calcium-dependent protein kinase (CDPK), calmodulin dependent protein kinase (CaMK), and CBL interacting protein kinase (CIPK) [36,37,38,39]. Proline-directed motif SP, or [pS/pTP]-type motif, is also an extremely common motif as a potential substrate for MAPK, CDPK, SnRK2, and RLK [36,37,38]. A subtype of motif SP, SXSP (SXpSP) [37], was significantly enriched in cold up-regulated phosphopeptides after 2 h of cold treatment. SXXD motif is acidic S-type targeted by SnRK1, CDPK, and casein kinase II [36,37].

### 2.3. Functional Analysis of the Cold-Responsive Proteome and Phosphoproteme Reveals Different Groups of Functional Terms

**Figure 6:**
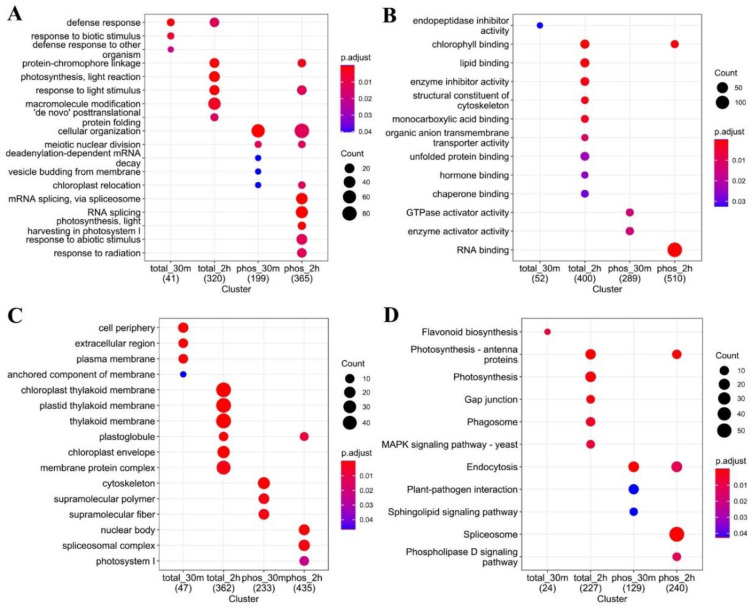
Functional enrichment of cold-responsive proteins and phosphoproteins using GO terms of biological process (**A**), molecular function (**B**), and cellular component (**C**), or using KEGG pathway terms (**D**).

We applied gene ontology (GO) enrichment analysis—in terms of biological process (BP), cellular component (CC), and molecular function (MF)—to cold-responsive proteins and phosphoproteins (proteins with cold-responsive phosphopeptides). Different GO terms were enriched between cold-responsive proteins and phosphoproteins, and between earlier responsive (30 min) and later responsive (2 h) ones (Figure 6). GO-BP enrichment analysis suggested that cold-responsive proteins were initially (30 min) enriched in biological processes involved in defense response and response to biotic stress, and then extended to protein-chromophore linkage, photosynthesis (light reaction), response to light stimulus, and macromolecular modification. However, for the cold-responsive phosphoproteome, cellular organization and meiotic nuclear division were the most significantly enriched GO-BP terms in both early and later cold-responsive phosphoproteome, and chloroplast relocation, mRNA splicing, photosynthesis (light harvesting), and response to abiotic stimulus terms were observed as the most enriched GO-BP terms in the 2 h cold-responsive phosphoproteome. For the GO-CC terms, the analysis identified cell periphery, extracellular region and plasma membrane as the most enriched terms for the early cold-responsive proteins, and thylakoid membrane, chloroplast envelope, plastoglobule and membrane protein complex as the most enriched ones in later cold-responsive proteins. However, for the phosphoproteome analysis, enriched GO-CC terms included cytoskeleton, supramolecular polymer in 30 min cold-responsive phosphoproteins, and nuclear body, spliceosome complex, photosystem I, and plastoglobule in 2 h cold-responsive phosphoproteins. In the GO-MF terms, RNA-binding stood out as the largest group of enriched terms in 2 h cold-responsive phosphoproteins.

KEGG pathway enrichment analysis also supported that cold-responsive proteins and phosphoproteins were enriched in different pathways. Early cold-responsive proteins were enriched in flavonoid biosynthesis, while later cold-responsive proteins were enriched in photosynthesis, phagosome, and gap junction pathways. Early cold-responsive phosphoproteins were associated with endocytosis, plant-pathogen interaction and sphingolipid signaling pathway, while later cold-responsive phosphoproteins were associated with endocytosis, spliceosome, photosynthesis, and phospholipase D signaling.

Interestingly, our global proteome analysis indicated that flavonoid biosynthesis was the only pathway enriched among early cold-responsive proteins (Figure 6D), consistent with a rapid increase in the flavonoid content and induction of expression of flavonoid biosynthetic genes in cold-treated maize [22,40]. In this study, three proteins involved in the flavonoid biosynthesis were up-regulated upon 30 min of cold stress (Table S2), including a chalcone synthase (P24825), a chalcone isomerase (B6TJA9), and an anthocyanidin synthase (P41213). In *Arabidopsis*, genetic evidence has supported that chalcone synthase and chalcone isomerase act as positive regulators of freezing tolerance [41]. 

### 2.4. Site-Specific Phosphorylation Modification during Cold Treatment

**Figure 7:**
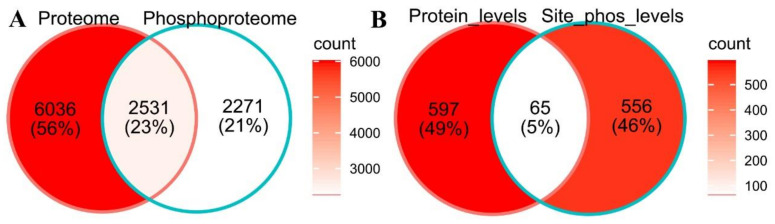
Venn diagrams showing the overlap between proteins observed in the proteome and in the phosphoproteome analyses (**A**), and between cold-responsive proteins at protein abundance levels and at site-specific phosphorylation levels (**B**) in maize seedlings during cold shock.

To determine whether the changes in phosphopeptide abundance were due to alterations in protein accumulation or because of changesat site-specific phosphorylation levels, we normalized the phosphopeptide levels to the corresponding protein abundance. Only approximately half of the proteins in the phosphoproteome were observed and quantified in the global proteome in this study (Figure 7). Normalized phosphopeptides were considered cold responsive at site-specific phosphorylation levels after 30 min or 2 h of cold treatment based on criteria of a fold-change of >1.4 or <0.71 and an adjusted *p*-value of less than 0.01. A total of 611 and 1025 phosphopeptides showed alterations at site-specific phosphorylation levels after 30 min and 2 h of cold treatment, respectively (Table S3). Interestingly, 662 and 621 proteins were cold responsive at protein abundance and site-specific phosphorylation levels, respectively, but only about 5% of them (65 proteins) were shared between them (Figure 7B, Tables S2 and S3), suggesting cold stress regulates many proteins only at site-specific phosphorylation levels.

### 2.5. Cold-Responsive Photosynthesis and Spliceosome Proteins

**Figure 8:**
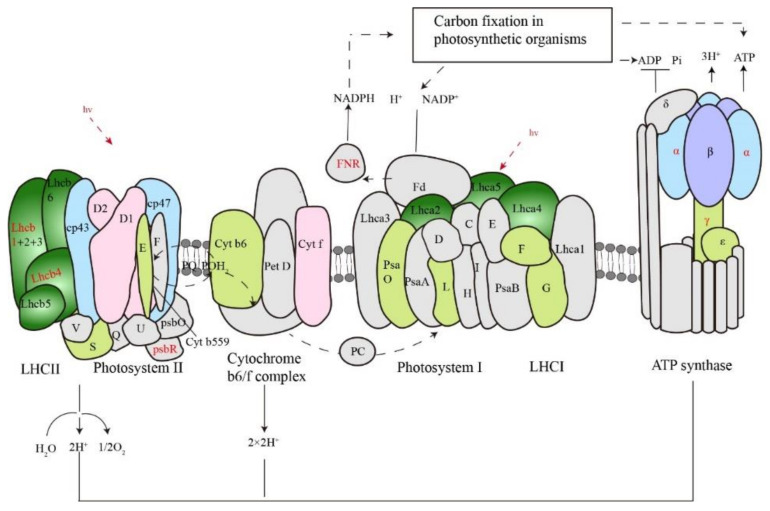
Schematic diagram of photosynthetic complexes on the thylakoid membrane. Cold-responsive proteins at protein abundance levels are shown in colors, while those unquantified or not-responsive are shown in gray. Proteins responsive to cold at site-specific phosphorylation levels are shown in red fonts.

GO and KEGG functional analyses indicated that the photosynthesis pathway is affected by cold response in maize, especially proteins involved in light capture and electron transfer reaction. There were no cold-responsive photosynthetic light-reaction proteins after 30 min of cold shock, but 32 cold-responsive ones after 2 h (Figure 8 and Table S4), all of which were down-regulated by cold. The cold-responsive proteins included components in the photosystem I and II, antenna proteins, the cytochrome b6/f complex, and the ATP synthase complex. Interestingly, a few proteins also showed alterations in site-specific phosphorylation, including Lhcb1, Lhcb4, photosystem II protein PsbR, ferredoxin-NADP^+^ reductase, and ATP synthase alpha and gamma subunits (Figure 8 and Table S4).

**Figure 9:**
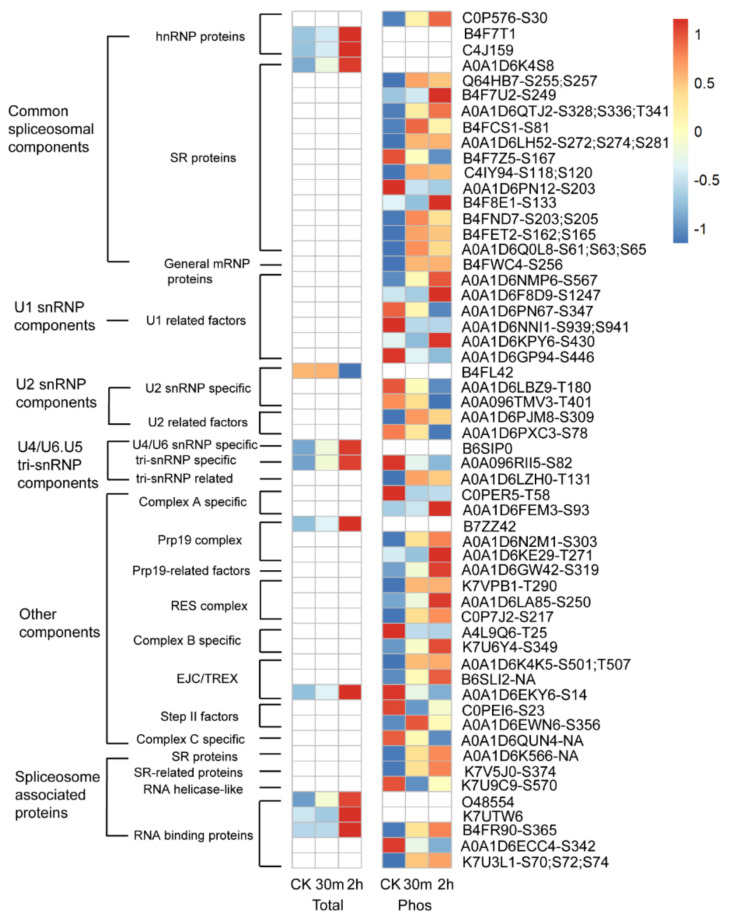
Expression profiles of cold-responsive spliceosome proteins at protein abundance (Total) or site-specific phosphorylation levels (Phos). Protein and phosphopeptide intensities were log2 transformed and scaled for display. Dark blue to dark red color gradient denotes lower to higher expression. One representative phosphopeptide of each phosphoprotein is shown. The accession numbers and the respective phosphosites are listed on the right of the panel. “NA” indicates the phosphosites were not confidently determined (ptmRS site probability < 75%).

Functional enrichment analysis indicated that spliceosome is a major pathway responsive to cold in maize. Splicing converts mRNA precursors into mature mRNAs by removing noncoding introns and joining protein-coding exons in eukaryotic organisms, and is accomplished by the spliceosome—an intricate complex macromolecular machine made of five small nuclear ribonucleoproteins (snRNPs), U1, U2, U4, U5, and U6 snRNPs, and several spliceosome-associated proteins (SAPs) [42,43]. Rapid and dynamic alternative splicing was reported in the cold response in *Arabidopsis* [44]. In this study, there were no cold-responsive spliceosome proteins at protein abundance levels within 30 min of cold shock, and there were 10 proteins up-regulated and one protein down-regulated after 2 h of cold treatment (Figure 9 and Table S4). In contrast to the relatively slow response at the protein abundance levels, 7 and 17 spliceosome proteins showed down- and up-regulation at site-specific phosphorylation levels upon 30 min of cold stress, respectively (Figure 9 and Table S4). Additionally, after two hours, 16 spliceosome proteins showed down-regulation and 32 proteins showed up-regulation at site-specific phosphorylation levels (Table S4).

### 2.6. Cold-Responsive Kinases and Transcription Factors

In this study, 31 kinases were found to be cold responsive within 30 min or 2 h of treatment in maize seedlings, including 7 kinases at the protein abundance levels (Table S5), 22 at the phosphopeptide levels, and 8 at site-specific phosphorylation levels (Table 1, Table 2 and Table S5). Interestingly, all the seven kinases were down-regulated after two hours of cold treatment, including two CDPKs, an aarF domain-containing kinases, a casein kinase II subunit alpha, a pyruvate dehydrogenase kinase, and a serine/threonine-protein kinase PBS1. At the site-specific phosphorylation level, three kinases in the MAPK cascade pathway, one CPK, two casein kinases 1 (CK1), and a serine/threonine-protein kinase were found to be responsive at site-specific phosphorylation levels. 

In *Arabidopsis*, the MAPK cascades MEKK1-MKK2-MPK4 and MKK4/5-MPK3/6 were demonstrated to positively and negatively regulate freezing tolerance, respectively [28,45]; in maize, the mitogen-activated protein kinase kinase kinase 1 (MAPKKK1), together with a mitogen-activated protein kinase kinase 1 (MEK1), and a mitogen-activated protein kinase 1/3 (MAPK1_3), were significantly altered at the site-specific phosphorylation level (Table 2 and Table S5), suggesting potential MAPK cascade pathways responsible for a cold adaptation in maize. 

As a class of well-characterized Ca^2+^ sensors, CPKs are serine/threonine protein kinases involved in plant stress response, including cold stress [46]. The rice OsCPK24 was demonstrated to positively regulate cold tolerance [47], and its ortholog in maize (CPK21) was found to be cold responsive in this study (Table 2).

In our study, 33 transcription factors were found to be cold responsive in maize seedlings, including 4 at the protein abundance levels, 30 at the phosphopeptide levels, and 16 at the site-specific phosphorylation levels (Table 1, Table 2, Tables S2 and S5 ). Three transcription factors increased in abundance after 2 h of cold treatment, including a basic leucine zip protein, a zinc-finger protein, and a heat shock transcription factor. A WRKY transcription factor protein (WRKY33) was down-regulated at 30 min, but recovered its abundance after 2 h of cold treatment. A total of 16 transcription factors were found to be cold responsive at the site-specific phosphorylation levels, including 11 bZIP proteins, a zinc finger protein, a homeobox-leucine zipper protein, a nuclear transcription Y (NF-Y) subunit beta protein, and a calmodulin-binding transcription activator (CAMTA). Four VIP transcription factors, three HY5 homologs, and one ABF were among the 11 cold-responsive bZIP transcription factors.

CAMTA1/2/3/5 are important regulators of cold tolerance in *Arabidopsis* by regulating the expression of key cold-responsive transcription factors CBFs [48,49,50], and they respond to cold stimuli at protein phosphorylation levels, but not at transcription or protein abundance levels [48,49]. In this study, we found that the phosphorylation of the CAMTA homologs in maize (A0A1D6IK52) were significantly altered without disturbing the protein abundance (Tables S2 and S3), suggesting similar functions of maize CAMTAs in cold response. 

The bZIP transcription factor Elongated hypocotyl 5 (HY5) serves as a central hub protein in transcriptionally regulating genes of multiple biological pathways including cold tolerance [51]. The activity of HY5 is controlled by its phosphorylation status [52]. We found that upon a short time of cold stress, the phosphorylation levels of three HY5 homologs (K7VAC7, K7VQH0, A0A1D6H3I5) in maize were all up-regulated (Table 2), suggesting roles in maize cold tolerance. 

## 3. Discussion

In this study, we performed a comprehensive proteome and phosphoproteome profiling of maize seedlings in their early response to rapid temperature drop. Over 8500 proteins and 19,300 distinct phosphopeptides were quantified, and over 1200 proteins were shown to be responsive to cold shock at protein abundance levels or site-specific phosphorylation levels within 2 h of cold shock, including many transcriptional factors and kinases whose homologs in *Arabidopsis* or rice are known to be involved in cold tolerance. 

Several lines of evidence suggest that maize and *Arabidopsis* share similarities in their early response to cold shock, especially at the phosphoproteome level. A rapid response at the phosphoproteome levels were observed in both maize and *Arabidopsis* in as early as 30 min. Additionally, after 2 h of cold shock, a total of 2024 phosphopeptides representing 1055 proteins were cold responsive in maize, while alterations of over 2038 phosphopeptides representing 1208 proteins at phosphopeptide levels were observed in *Arabidopsis* [32]. KEGG and GO enrichment analysis suggest that photosynthesis, spliceosome, endocytosis, and response to biotic and abiotic stresses were the major pathways associated with rapid cold response in maize, in agreement with similar studies in *Arabidopsis* [31,32]. Three of the four enriched phosphorylation motifs of cold up-regulated phosphopeptides in maize—which included RXXS, SP, and SXXD—were previously reported in similar studies in *Arabidopsis* [31,32]. In addition, known cold-signaling proteins in *Arabidopsis*, such as MAPK signaling cascade proteins, were also identified in this study. 

Maize showed much more rapid responses at the global proteome level, distinct from that in *Arabidopsis* when exposed to a sudden drop in temperature. In this study, 612 proteins (out of 8567 proteins) showed a significant change within 2 h of cold treatment, distinct from the few proteins (7 out of 6733) reported in a similar study in *Arabidopsis* [32]. Different fold-change thresholds were used to identify cold-responsive proteins in the two studies, as ratios are underestimated in MS^2^-based TMT quantitative methods because of co-eluting interfering peptides [34,53].

Chilling stress damages photosystems in maize [17]; we provide evidence that photosynthesis proteins drop much earlier than previously reported in maize. Previous studies showed that photosynthetic light-reaction proteins decreased after long durations of cold treatment [17], such as 12 h of cold treatment at 4 °C [54]. In this work, we showed that photosynthesis (light reaction) proteins were mostly unchanged within 30 min of cold shock, but 32 light-reaction proteins dropped in abundance after 2 h of treatment, including components in light harvesting, photosystem I and II, electron transfer, and ATP synthase complex. The abundance of photosynthesis proteins is balanced by their biosynthesis and degradation. Protease activities were detected in the chloroplast, such as a metalloprotease Fish Protease 6 (AtFtsH6), which is responsible for the degradation of LHCb1 and LHCb3 under high light and senescence [55]. Recently, a ubiquitin-dependent degradation process was observed during chloroplast degradation, and a degradation of intra-chloroplast proteins RbcL and AtpB was mediated by a chaperone-like CDC48 complex [56]. Interestingly, when the global proteome data were searched against the maize database using ubiquitination of lysine as a variable modification, 11 cold down-regulated photosynthesis proteins were found to contain ubiquitin sites, including six chlorophyll a-b binding proteins, three ATP synthase subunits (alpha, beta, and gamma) and one cytochrome f proteins (Table S6). Further analysis using enriched ubiquitinated peptides would uncover more ubiquitination-related molecular events during cold shock, and genetic evidence is needed to support the roles of ubiquitination in cold-regulated degradation of photosynthesis light-reaction proteins. It should be noticed that temperatures at 4 °C could cause irreversible cellular and tissue damages in maize seedlings [1,17], and a sudden temperature drop from 25 °C to 4 °C might lead to unpredicted tissue and cellular injuries for seedlings. Future studies are needed to profile proteome and phosphoproteome of maize seedlings to identify changes in the abundance and phosphorylation of photosystem proteins under a gradual temperature decrease condition, and to compare photosystem II efficiency between the conditions of under a sudden temperature drop and a gradual temperature drop.

Enhanced alternative splicing is observed in plants under stress [57,58], and our study suggests that an increase in the protein abundance and alteration of the phosphorylation of spliceosome proteins likely contribute to increased alternative splicing events during cold shock in maize. Alternative splicing generates multiple mRNA transcripts from a single mRNA precursor, expands the diversity of transcriptome and proteome, and thus, changes the activity, subcellular localization, and protein–protein interaction of the protein isoforms [57,58]. Over 2400 genes were altered at the alternative splicing levels by cold treatment in *Arabidopsis*, and one of the cold-responsive spliceosome component *U2B’-like* gene was shown to regulate freezing tolerance in *Arabidopsis* [44]. Over 48,000 mRNA isoforms were found in maize, and their levels were regulated by developmental stages and growth conditions (drought or well-watered) [59]. In addition, high temperatures enhance alternative RNA splicing events in maize, especially for genes encoding the spliceosome components [60]. In this study, 10 spliceosome proteins showed an increase in the protein abundance, and 48 proteins were altered at site-specific phosphorylation levels; these cold-responsive spliceosome proteins were from all the major spliceosome components, including the five U snRNPs, common spliceosome components, spliceosome associated proteins, and other snRNP components. Reversible protein phosphorylation is a key mechanism in regulating the activity of splicing factors in mammals [61,62], but were hardly studied in plants [63]. Further work will be needed to uncover the kinases and phosphatases responsible for the reversible phosphorylation of spliceosome proteins and their roles during cold stress in maize.

To our knowledge, this is the first comprehensive proteome and phosphoproteome profiling of the rapid molecular events (within 2 h) in maize seedling under a sudden temperature drop, which would be a much-needed resource for uncovering the molecular basis of the cold adaptation in maize.

## 4. Materials and Methods

### 4.1. Plant Materials and Growth Conditions

Maize (*Zea mays* L.) cv. B73 seeds were surface sterilized with 2% sodium hypochlorite solution for 30 min, washed 5 times with distilled water, and sown in Lambert LM-GPS soil. Seedlings were grown in a growth room at 25 °C under daily light exposure of 14 h with 60 μmol m^−2^ s^−1^ white light. After two weeks of growth, seedlings at five-leaf stage were transferred into a precooled bench-top temperature chamber XT5438 (Xutemp, Hangzhou, China) with approximately 1.0 μmol m^−2^ s^−1^ white light for cold treatment. The above-ground stem and leaf tissues were collected after 30 min and 2 h treatments at 4 °C, frozen in liquid nitrogen, and stored at −80 °C for further experiments.

### 4.2. Protein Extraction, Trypsin Digestion, and TMT Labeling

Proteins were extracted from frozen tissues using modified phenol-methanol protocol [64]. Approximately 0.15 g liquid-nitrogen-ground tissue powder was thoroughly mixed with 450 μL extraction buffer (100 mM Tris-HCl (pH 8.0), 2% SDS, 10 mM EDTA, 5 mM EGTA, 1 mM PMSF, 2% 2-mercaptoethanol), and supplemented with a protease and phosphatase inhibitor cocktail (Thermo Fisher Scientific, Waltham, MA, USA). The samples were then heated for 10 min at 65 °C, and centrifuged at 20,000× *g* for 20 min. The supernatant was mixed with an equal volume of ice-cold Tris-saturated phenol (pH 8.0) and centrifuged at 20,000× *g* for 15 min at 4 °C to separate phenol and aqueous phases. The upper aqueous phase was removed, leaving the interface intact, and the phenol phase was extracted twice with 50 mM Tris-HCl, pH 8.0, mixed with 5 volumes of cold 0.1 M ammonium acetate in methanol, and kept at −40 °C overnight to precipitate proteins. After centrifugation at 20,000× *g* for 20 min, the protein pellet was washed once with 1 mL cold 0.1 M ammonium acetate in methanol and three times with 1 mL cold methanol. Afterwards, the protein pellet was air-dried and dissolved in 250 μL 8M urea, and the protein concentration was determined with a Protein Assay kit (Bio-Rad Laboratories, Hercules, CA, USA), using BSA as a standard. Then, protein samples were reduced with 20 mM Tris-(2-carboxyethyl)-phosphine (TECP) for 60 min at 30 °C and then alkylated with 30 mM iodoacetamide at 25 °C for 40 min in the dark. Samples were diluted to a final concentration of 1.6 M urea with 50 mM ammonium bicarbonate before trypsin digestion at a 1:25 enzyme:substrate ratio. The digestion was performed at 37 °C for 16 h at 800 rpm, and was terminated after trifluoroacetic acid (TFA) was added to a final concentration of 1%. The resulting peptides were desalted on a Strata-X 33 μm polymeric reversed phase column (Phenomenex, Torrance, CA, USA) and resuspended in 50 mM triethylammonium bicarbonate (TEAB) buffer. Each peptide sample was combined with a respective 9-plex TMT reagent (control samples: 126, 127N, 127C; 30 min-treated samples: 129N, 129C, 130N; 2-hr-treated samples: 130C, 131N, 131C) and incubated for 1 h at room temperature. The reaction was stopped by an addition of 5% hydroxylamine to a final concentration of 1% and incubated for further 15 min. TMT-labeled samples were combined for high-pH fractionation.

### 4.3. Peptide Fractionation and Phosphopeptide Enrichment

To reduce peptide complexity, samples were separated by basic reversed-phase chromatography. Multiplexed TMT-labeled samples were combined and separated on a Waters Acquity BEH C18 column (1.7 μm particle size, 2.1 mm ID, 100 mm length) using H class UPLC system (Waters) at a flow rate of 300 μL/min. TMT-labeled peptides were separated by a linear gradient from 2 to 8% buffer B (100% acetonitrile, 5 mM ammonium hydroxide) in 1.5 min, to 24% buffer B in 15.5 min, to 32% buffer B within 4 min, then from 32% to 70% buffer B in 1 min. The buffer A is 5 mM ammonium hydroxide solution. A total of 30 fractions were collected, combined into 15 fractions for global proteome analysis, or combined into 7 fractions for phosphopeptide enrichment.

Enrichment of phosphorylated peptides was conducted using immobilized metal affinity chromatography (IMAC) as described. Briefly, fractionated samples were incubated with gallium chloride-charged chelating Sepharose fast flow slurry (GE Healthcare, Piscataway, NJ, USA) for 30 min at 800 rpm. Sepharose resins were then washed 5 times with wash buffer (80% acetonitrile and 0.1% TFA), and phosphorylated peptides were eluted three times in elution buffer (5% ammonium hydroxide, 50% acetonitrile). Eluted fractions were pooled and freeze-dried in vacuum and were then reconstituted in 0.1% TFA for nano-LC-MS/MS analysis.

### 4.4. LC-MS/MS Analysis

Samples were analyzed on an Ultimate 3000 nano UHPLC system (Thermo Scientific, Waltham, MA, USA) equipped with a trapping column (PepMap C18, 100 Å, 100 μm × 2 cm, 5μm, Thermo Scientific) and an analytical column (PepMap C18, 100 Å, 75 μm × 50 cm, 2μm, Thermo Scientific), coupled online to a Q Exactive HF hybrid mass spectrometer (Thermo Scientific) equipped with a Nanospray Flex Ion Source (Thermo Scientific). Each fraction of TMT-labeled peptides (1 μg) was separated by a binary buffer system of buffer A (0.1% formic acid) and buffer B (80% acetonitrile, 0.1% formic acid). For separation of peptides of the global proteome, samples were equilibrated in 5% buffer B, then eluted in a linear gradient from 8 to 11% buffer B within 6 min, to 43% buffer B within 106 min, to 90% buffer B within 6 min. For separation of phosphopeptide samples, samples were first equilibrated in 5% buffer B, then eluted in a linear gradient from 8 to 11% buffer B within 5 min, to 43% buffer B within 67 min, to 90% buffer B within 6 min. The column oven was both set at 60°C and the flow rate was 300 nL/min.

The scanning parameters for MS were the same for both total peptides and phosphopeptides. The full scan was performed between 350–1650 m/z at the resolution 120,000 at 200 m/z, with the automatic gain control target at 3e6. The MS/MS scan was operated with HCD in top 12 mode using the following settings: resolution 45,000 at 200 m/z; automatic gain control target at 1e5; normalized collision energy at 32%; isolation window of 1.2 Th; charge state exclusion: unassigned, 1, >7; dynamic exclusion 30 s.

### 4.5. LC-MS/MS Data Analysis and Bioinformatics Analysis

Raw instrument files were processed using Proteome Discoverer version 2.4.0.305 (Thermo Scientific). Protein identification was performed using the SEQUEST HT search engine with UniProt maize proteome database (a total of 99,253 entries as of 28 May 2021). Searches were configured with static modifications for the TMT reagents (+229.163 Da) on lysines and N-termini, carbamidomethylation on cysteines, dynamic modifications for oxidation of methionine residues and acetylation of protein N-termini, precursor mass tolerance of 10 ppm, fragment mass tolerance of 0.02 Da, and trypsin cleavage (max 2 missed cleavages). For the identification of phosphopeptides in IMAC-enriched samples, phosphorylation of serine, threonine, and tyrosine was set as an additional dynamic modification, and PhosphoRS mode was on in ptmRS node of Proteome Discoverer. Searches used a reversed sequence decoy strategy to control peptide false discovery and used Percolator to validate identifications. The identification of PSM, peptides, and proteins was performed at the FDR cutoff of 1% level (q scores < 0.01). Normalization was applied for the grand total reporter ion intensity for each channel within the 9-plex experiment. This corrects for small sample loading and labeling reaction efficiency differences. Statistical analyses were performed in an R environment using the limma package from Bioconductor (http://www.bioconductor.org/ accessed on 4 June 2022). Differentially abundant proteins were filtered for an average fold-change of >1.3 or <0.770, with *p*-values < 0.01 after adjusted for multiple testing correction by false discovery rate (FDR) (Benjamini–Hochberg). Differentially abundant phosphopeptides showing *p*-values < 0.01 and an average fold change of >1.4 or <0.714 were considered for cold-responsive phosphopeptides.

The PCA analysis was performed using the PCAtools packages in the R environment, and the log2 transformed intensity values of all proteins/phosphopeptides which were shared among the 9 samples were used as features of PCA. GO and KEGG enrichment analyses were performed on the cold-responsive proteins or proteins with cold-responsive phosphopeptides using ClusterProfiler in the R environment, with FDR < 0.05 considered as over-representative terms. GO annotations were downloaded from UniProt database (release of May 2021). KEGG pathway mapping of maize proteins were conducted online with BlastKOALA v2.2 (KEGG Orthology And Links Annotation) against the “species_eukaryotes” database (https://www.kegg.jp/blastkoala/, accessed on 29 March 2022) [65]. For motif analysis, 15-bp amino acid sequences centered on the cold-responsive phosphosites were submitted to MoMo (Modification Motifs, https://meme-suite.org/meme/tools/momo, accessed on 7 April 2022) [35] and processed using the settings as Width = 15, Minimum number = 25, and *p*-value threshold = 0.000001. Venn diagrams were plotted using the ggplot2 [66] and ggVennDiagram [67] R packages. Heatmap was created with pheatmap R packages. Schematic diagram and workflow were manually drafted using Adobe Illustrator 2021.

## Figures and Tables

**Table 1:** Cold-responsive kinases and transcriptional factors (at the protein abundance levels). “Ratio” refers to protein abundance ratio of treated/control. “*p*-value” refers Benjamini–Hochberg adjusted *p*-value.

Accession	Ratio (30 min)	*p* Value (30 min)	Ratio (2 h)	*p* Value (2 h)	Description
Kinases					
A0A1D6FNF9	0.96	7.27 × 10^−1^	0.76	8.14 × 10^−3^	ABC1-like kinase
A0A1D6HW78	0.87	1.34 × 10^−1^	0.71	1.47 × 10^−3^	ABC2 homolog 13
A0A1D6JD09	1.03	7.53 × 10^−1^	0.74	2.67 × 10^−3^	Calcium-dependent protein kinase
B4F9P5	1.09	1.95 × 10^−1^	0.76	1.09 × 10^−3^	Protein-serine/threonine kinase
B4FF99	0.76	1.32 × 10^−2^	0.70	8.59 × 10^−4^	Calcium-dependent protein kinase 7
B6SVK8	1.02	8.50 × 10^−1^	0.74	5.73 × 10^−3^	Serine/threonine-protein kinase NAK
P28523	0.91	3.87 × 10^−1^	0.73	7.04 × 10^−3^	Casein kinase II subunit alpha
Transcription factors					
A0A1D6JVI5	1.02	8.94 × 10^−1^	2.88	2.38 × 10^−5^	bZIP transcription factor 16
B4FIJ2	1.15	8.66 × 10^−2^	1.35	1.42 × 10^−3^	Zinc ion binding
K7V3U5	0.64	6.49 × 10^−3^	0.99	9.31 × 10^−1^	WRKY DNA-binding domain protein
K7V9Y4	1.06	4.10 × 10^−1^	1.31	1.87 × 10^−3^	HSF28 HSF type transcription factor

**Table 2:** Cold-responsive kinases and transcriptional factors (at site-specific phosphorylation levels). “Ratio” refers to phosphorylation level ratio of treated/control. “*p*-value” refers Benjamini–Hochberg corrected *p*-value.

Accession	Ratio (30 min)	*p* Value (30 min)	Ratio (2 h)	*p* Value (2 h)	Phos-Site	Description
Kinases						
A0A1D6F0U9	1.35	3.17 × 10^−2^	2.07	2.71 × 10^−4^	S498/T501	MAPK
A0A1D6G325	0.64	6.58 × 10^−4^	0.66	7.95 × 10^−4^	S279	Casein kinase family protein
A0A1D6GJU6	0.78	8.83 × 10^−3^	0.63	2.71 × 10^−4^	S115	MAPKKK1
A0A1D6GJU6	0.75	1.77 × 10^−2^	0.63	9.43 × 10^−4^	S200	MAPKKK1
A0A1D6ICZ3	0.64	9.04 × 10^−4^	0.63	5.87 × 10^−4^	T52	CDPK8
A0A1D6MYW4	0.68	2.64 × 10^−3^	0.68	2.22 × 10^−3^	S253	CPK21
A0A1D6P094	1.68	2.92 × 10^−3^	1.03	8.48 × 10^−1^	S436	Protein kinase
B4FBJ3	0.77	8.28 × 10^−3^	0.64	3.24 × 10^−4^	S464	Casein kinase 1
O49975	0.51	1.42 × 10^−4^	0.57	3.52 × 10^−4^	T30	MEK1
Transcription factors						
A0A1D6EHU0	0.67	6.5 × 10^−2^	0.54	6.5 × 10^−3^	S130	VIP1 transcription factor
A0A1D6EHU0	0.34	1.6 × 10^−4^	0.53	2.5 × 10^−3^	S31	VIP1 transcription factor
A0A1D6EK20	0.71	3.6 × 10^−3^	0.74	4.7 × 10^−3^	S930	RNA binding family protein
A0A1D6EK20	0.58	2.5 × 10^−4^	0.72	2.7 × 10^−3^	S949	RNA binding family protein
A0A1D6ELA3	1.15	1.8 × 10^−1^	1.40	5.8 × 10^−3^	S180	G-box-binding factor 1
A0A1D6H3I5	1.27	5.4 × 10^−2^	1.43	6.3 × 10^−3^	S83	HY5 transcription factor homolog
A0A1D6IJ69	1.56	6.9 × 10^−4^	1.46	1.3 × 10^−3^	S205	NF-Y subunit B-3
A0A1D6IK52	0.81	5.2 × 10^−2^	0.70	3.3 × 10^−3^	S172	CAMTA 2
A0A1D6JVI5	0.93	7.0 × 10^−1^	0.46	8.8 × 10^−4^	S203	bZIP transcription factor 16
A0A1D6JVI5	0.95	6.4 × 10^−1^	0.41	6.9 × 10^−5^	S148; S151	bZIP transcription factor 16
A0A1D6JVI5	1.06	5.4 × 10^−1^	0.38	3.3 × 10^−3^	S290	bZIP transcription factor 16
A0A1D6K5M3	1.57	4.9 × 10^−3^	1.52	4.9 × 10^−3^	S121	NF-Y subunit B-2
A0A1D6K5M3	1.47	3.5 × 10^−3^	1.36	6.9 × 10^−3^	S212	NF-Y subunit B-2
A0A1D6K5M3	1.53	2.1 × 10^−2^	1.61	7.9 × 10^−3^	S210; S213	NF-Y subunit B-2
A0A1D6MZQ6	1.39	1.1 × 10^−2^	1.45	4.5 × 10^−3^	S124	VIP1 transcription factor
A0A1D6PUS5	1.65	3.2 × 10^−3^	1.24	7.6 × 10^−2^	S89	Homeobox-leucine zipper protein MERISTEM L1
B4F937	1.51	3.5 × 10^−3^	1.33	1.4 × 10^−2^	S147	G-box binding factor
B4FWJ9	0.53	2.7 × 10^−4^	0.47	1.1 × 10^−4^	S139	bZIP transcription factor
B4FWJ9	0.60	8.5 × 10^−5^	0.66	2.7 × 10^−4^	S31	bZIP transcription factor
C4J4L1	0.69	7.5 × 10^−3^	0.69	5.2 × 10^−3^	T134	ABI5-like protein 2
K7TX82	0.78	1.7 × 10^−2^	0.66	8.9 × 10^−4^	S192	VIP1 transcription factor
K7VAC7	1.07	6.9 × 10^−1^	1.93	1.6 × 10^−3^	S132	HY5 transcription factor homolog
K7VQH0	1.12	3.4 × 10^−1^	1.50	4.7 × 10^−3^	S30	HY5 transcription factor homolog

## Data Availability

The mass spectrometry proteomics data would have been deposited to the ProteomeXchange Consortium (http://proteomecentral.proteomexchange.org, accessed on 5 June 2022) via the iProX partner repository [68] with the dataset identifier PXD034262.

## Supplementary Materials

The following supporting information can be downloaded at: https://www.mdpi.com/article/10.3390/ijms23126493/s1.
